# Prevalence of Contact Allergy to Colophonium in Dermatitis Patients: A Systematic Review and Meta‐Analysis

**DOI:** 10.1111/cod.70153

**Published:** 2026-04-05

**Authors:** Kian Karimian, Daniel Isufi, Mikkel Bak Jensen, Jeanne Duus Johansen, Jakob Ferløv Baselius Schwensen

**Affiliations:** ^1^ National Allergy Research Centre, Department of Dermatology and Allergy Herlev and Gentofte Hospital Copenhagen Denmark; ^2^ Department of Dermatology and Allergy Herlev and Gentofte—Copenhagen University Hospital Copenhagen Denmark; ^3^ Institute of Clinical Medicine, Faculty of Health and Medical Sciences, University of Copenhagen Copenhagen Denmark

**Keywords:** colophonium, colophony, contact allergy, dermatitis, gum rosin, rosin, tall oil rosin, wood rosin

## Abstract

Colophonium is present in many consumer and industrial products, including medical devices and cosmetic products. Due to its extensive use, there is an inherent risk of developing a contact allergy to it. Previous studies have shown that the prevalence rate of contact allergy to colophonium is around 1%–2%. This meta‐analysis aimed to estimate the current prevalence of colophonium contact allergy in dermatitis patients. Two authors independently searched PubMed, Embase and Web of Science for studies reporting the prevalence of contact allergy in dermatitis patients with inception through 30 June 2025. A proportion meta‐analysis was conducted to estimate the pooled prevalence of colophonium contact allergy. Seventy‐three studies involving 459 757 patients with dermatitis were included. The pooled prevalence of colophonium contact allergy was 3.54% (95% CI, 3.01–4.16) with an *I* ^2^ of 98.6% (95% CI, 98.5–98.7) and a clinical relevance of 39.42% (95% CI, 37.94–40.92). Significant sensitization rates were observed in different geographic regions, with the highest prevalence found in Southeast Asia (6.83%). The prevalence rate was 3.31% among children and 1.31% among adults; however, these differences were not statistically significant. The prevalence rate was 4.74% for patients with AD and 4.87% for patients without AD, with no statistically significant difference. Colophonium sensitization has remained stable in recent decades, highlighting the substance's widespread presence in products. This emphasizes the need for prevention and better registration.

**Abbreviations**
AXIS: appraisal tool for cross‐sectional studies
BOP: balsam of Peru
CI: confidence interval
FM1: fragrance mix 1
FM1/2: fragrance mix 1/2
FM2: fragrance mix 2
INCI: International Nomenclature of Cosmetic Ingredients
*n*
: number
PPT: positive patch test
PRISMA: Preferred Reporting Items for Systematic Reviews and Meta‐analyses
PROSPERO: International Prospective Register of Systematic Reviews

## 1 Introduction

Colophonium, also known as rosin, gum rosin, wood rosin, tall oil rosin or colophony, is a natural resin derived from pine trees. It is widely used for its adhesive properties [1]. Chemically, it is a complex mixture of resin acids and other constituents. Some constituents, particularly oxidation products, can act as sensitizing agents themselves [2]. Colophonium is present in numerous consumer and industrial products, including medical devices, cosmetics, inks and sports equipment [3]. In cosmetics, the International Nomenclature of Cosmetic Ingredients (INCI) requires use of the term ‘*Colophonium’*, whereas other synonyms are common in non‐cosmetic products, which complicates epidemiological surveillance. Colophonium is a common occupational allergen in the wood, paper, electronics and metalworking industries [2].

Colophonium is included in baseline patch test series worldwide at a concentration of 20% petrolatum (pet.) and is also part of the Thin‐layer Rapid Use Epicutaneous (TRUE) Test system [4] and the European Baseline Series (EBS) [5]. Although contact allergy to colophonium has been documented for decades, prevalence estimates vary considerably, ranging from 1.4% to 4.02% in studies [6, 7, 8]. These variations reflect differences in study populations, methodologies and regional contexts.

Despite extensive documentation, the global prevalence of contact allergy to colophonium remains poorly reported. This study aims to provide a comprehensive understanding of the prevalence of contact allergy to colophonium in patients with dermatitis according to age, geography, time period, atopic dermatitis (AD) status and clinical relevance.

## 2 Methods

Before initiation, a study protocol was registered on the Prospective Register of Systematic Reviews (PROSPERO; CRD420251062433). The study adhered to the Preferred Reporting Items for Systematic Review and Meta‐Analysis (PRISMA) guidelines [9].

### 2.1 Literature Search

Three databases (PubMed, Embase and Web of Science) were searched from inception through 30th June 2025 using the search strategy available in Table S1. All titles and abstracts were extracted and compiled into the web‐based screening tool Rayyan [10], and duplicates were manually removed.

### 2.2 Inclusion and Exclusion Criteria

Two authors (K.K. and D.I.) independently assessed studies for eligibility. Inclusion criteria were: (I) Original studies, (II) written in any language, (III) report the number of dermatitis patients undergoing consecutive patch testing and (IV) the number of positive patch tests (PPTs) to colophonium.

Exclusion criteria were: (I) grey literature (i.e., letters and conference abstracts) and (II) studies not conducted in dermatitis patients (i.e., general population). If publications involving the same population were retrieved, the most comprehensive report was selected for inclusion. When eligibility could not be determined from the title or abstract alone, the study was retained for full‐text assessment.

### 2.3 Data Extraction and Quality Assessment

Data were extracted by K.K. and D.I. into pre‐defined tables including information on the author surname, publication year, study period, country, concentration, vehicle, number of PPTs, sex distribution (male, %), age (mean, standard deviation [SD]), patients with AD (%) and clinically relevant PPTs (%). The study quality of cross‐sectional studies was assessed using the Appraisal tool for Cross‐Sectional Studies (AXIS) [11].

### 2.4 Statistical Analysis

Statistical analyses were performed using R in RStudio (version 2024.04.2, build 764). Pooled prevalence estimates and 95% confidence intervals (CIs) were calculated using a random‐effects model with inverse variance weighting. The DerSimonian–Laird estimator was used to estimate between‐study heterogeneity for tau^2^, while the Jackson method was employed to calculate CIs for both tau^2^ and tau. The *I* ^2^ statistic, which calculates the percentage of total variation that can be attributed to heterogeneity rather than chance, was derived from the Q statistic. All proportions were logit‐transformed prior to meta‐analysis to stabilize the variance and minimize the influence of extreme proportions. For individual studies, 95% CIs were calculated using the Clopper–Pearson exact method. Concomitant reactions were defined as the proportion of colophonium‐positive patients who also reacted to other allergens. Exact 95% CIs were calculated using binomial tests.

Data were extracted separately from studies that reported multiple concentrations for the same vehicle. As a result, the same author and year may appear multiple times in the analyses. If separate extraction was not an option, the data were grouped and included in the overall analysis used to categorize each allergen but excluded from the sub‐analyses.

## 3 Results

### 3.1 Qualitative Assessment of the Included Studies

#### 3.1.1 Eligible Studies

In total, 2300 articles (PubMed = 513, Embase = 1253 and Web of Science = 534) were included. After removing duplicates, a total of 1323 non‐duplicate articles were screened based on title and abstract. Of these, 90 were included for full‐text assessment. Based on the full‐text articles, 17 were excluded with reasons yielding 73 articles included in the meta‐analysis (Figure S1).

#### 3.1.2 Characteristics of the Included Studies

A total of 73 studies [7, 12, 13, 14, 15, 16, 17, 18, 19, 20, 21, 22, 23, 24, 25, 26, 27, 28, 29, 30, 31, 32, 33, 34, 35, 36, 37, 38, 39, 40, 41, 42, 43, 44, 45, 46, 47, 48, 49, 50, 51, 52, 53, 54, 55, 56, 57, 58, 59, 60, 61, 62, 63, 64, 65, 66, 67, 68, 69, 70, 71, 72, 73, 74, 75, 76, 77, 78, 79, 80, 81, 82, 83] were included, comprising 459 757 patients. Of these, 118 465 (25.77%) were males. The mean age across studies was 38.6 ± 12.8 years (*n* = 17 studies [13, 20, 23, 31, 35, 37, 39, 52, 54, 58, 69, 70, 71, 76, 77, 81, 83]). The number of patients with AD was reported in 36 studies [7, 13, 14, 16, 19, 20, 23, 27, 28, 29, 33, 34, 36, 40, 44, 45, 46, 51, 52, 54, 56, 57, 58, 61, 66, 68, 69, 70, 72, 73, 74, 75, 76, 77, 79, 83] yielding 46 432 (10.10%) patients with AD. The studies were conducted in Europe (*n* = 33 studies [7, 14, 18, 21, 27, 30, 33, 34, 35, 36, 41, 42, 43, 44, 45, 46, 48, 51, 55, 57, 59, 61, 64, 65, 68, 70, 73, 74, 77, 78, 79, 80, 81]), Middle East (*n* = 11 studies [15, 17, 19, 22, 23, 28, 39, 49, 71, 75, 76]), North America (*n* = 6 studies [12, 38, 53, 60, 63, 72]), South America (*n* = 2 studies [47, 50]), East Asia (*n* = 7 studies [13, 26, 37, 52, 54, 56, 62]), Southeast Asia (*n* = 9 studies [16, 20, 31, 32, 66, 67, 69, 82, 83]), Oceania (*n* = 4 studies [24, 25, 29, 40]), and Africa (*n* = 1 study [58]) (Table 1). Based on the AXIS assessment, most studies were marked as having an acceptable risk of bias (Table S2).

**TABLE 1:** Characteristics of the included studies.

First author surname (year)	Study period (year)	Country	Conc. (%) and vehicle	Patients (*n*)	Male (n)	Male (%)	Age (median [IQR])	Age (mean ± SD)	Atopic dermatitis (*n*)	Atopic dermatitis (%)	Atopic dermatitis positive (*n*)
Goon (2003) [82]	1999–2000	Singapore	20	1722	NA	NA	NA	NA	NA	NA	NA
Piaserico (2004) [81]	1997–2001	Italy	NA	1444	464	32.1	NA	72.7 ± 5.7	NA	NA	NA
Marinovic‐Kulisic (2004) [80]	1998–2003	Croatia	20	6341	NA	NA	NA	NA	NA	NA	534
Uter (2005) [79]	2002–2003	12 European countries	20	10511	3899	37.1	NA	NA	1892	18	NA
Bruynzeel (2005) [78]	1996–2000	8 European countries	20	26210	9089	34.68	NA	NA	NA	NA	NA
Machovcova (2005) [77]	1997–2001	Czech Republic	20	12058	4416	36.6	NA	41.7 ± 12.6	2062	17.1	NA
Kashani (2005) [76]	2002–2004	Iran	NA	250	60	24	NA	32.7 ± 13.1	89	35.6	NA
Magen (2006) [75]	2002–2005	Israel	NA	864	317	36.7	NA	NA	122	14.1	NA
Oppel (2006) [74]	2004–2004	Germany	NA	9948	3773	37.93	NA	NA	1671	16.8	NA
Devos (2008) [73]	NA	Belgium and Netherlands	NA	138	NA	NA	NA	NA	46	33.3	NA
Zug (2008) [72]	2001–2004	Canada and United States	20	391	137	35.04	NA	NA	157	40.2	NA
Ertam (2008) [71]	2000–2005	Turkey	NA	3017	1042	34.5	NA	40.38 ± 14.69	NA	NA	NA
Bordel‐Gomez (2009) [70]	2000–2005	Spain	20	1092	419	38,4	41.4 (1–90)^a^	41.4 ± 16.5	164	15	NA
Disphanurat (2010) [69]	2008–2009	Thailand	NA	157	45	28.7	NA	40.6 ± 15.2	11	7	NA
Heine (2006) [68]	1998–2003	3 European countries	20	24283	9098	37.47	NA	NA	9020	37.15	NA
Goon (2006) [67]	1986–2003	Singapore	20	2340	NA	NA	NA	NA	NA	NA	NA
Bajaj (2007) [66]	1997–2006	India	20	1000	566	56.6	35,9 (8–87)^a^	NA	76	7.6	NA
Lindberg (2007) [65]	1991–2001	Sweden	20	7470	2690	36.01	NA	NA	NA	NA	NA
Nardelli (2008) [64]	1990–2005	Belgium	NA	10128	3491	34.5	NA	NA	NA	NA	NA
Tudela (2008) [63]	1970–2002	Canada and United States	20	NA	NA	NA	NA	NA	NA	NA	NA
Lam (2008) [62]	1995–1999	Hong Kong	20	2585	1050	40.62	NA	NA	NA	NA	NA
Czarnobilska (2009) [61]	2007–2007	Poland	20	229	NA	NA	NA	NA	229	100	100
Wetter (2010) [60]	2000–2007	United States	20	945	121	12.8	53.4 (6–91)^a^	NA	NA	NA	NA
Garg (2009) [59]	2002–2007	United Kingdom	20	2076	701	33.77	NA	NA	NA	NA	NA
Bilcha (2010) [58]	2007–2008	Ethiopia	NA	514	171	33.27	36.3 (18–76)^a^	36.3 ± 12.1	8	1.5	NA
Beliauskiene (2011) [57]	2006–2008	Lithuania	20	816	215	26.3	46 (31–57)	NA	144	17.9	67
Dou (2011) [56]	1990–2009	China	20	1858	399	21.5	NA	NA	212	11.4	NA
Isaksson (2011) [55]	2006–2007	Sweden	20	2818	993	35.24	NA	NA	NA	NA	NA
Cheng (2011) [54]	2001–2006	China	20	1354	363	26.8	38.0 (5–81)^a^	38.0 ± 14.1	63	4.65	NA
Landeck (2011) [53]	1990–2006	United States	NA	1247	373	29.91	NA	NA	NA	NA	NA
Yin (2011) [52]	2004–2009	China	20	2758	1136	41.2	38.5 (17–68)^a^	38.5 ± 12.4	215	7.8	NA
Thyssen (2012) [51]	1984–2010	Denmark	20	15641	5681	36.32	NA	NA	1218	7.79	41
Rodrigues (2012) [50]	2003–2010	Brazil	20	1406	426	30.3	NA	NA	NA	NA	NA
Almogren (2012) [49]	2008–2010	Saudi Arabia	NA	196	NA	NA	NA	NA	NA	NA	NA
Uter (2012) [48]	2007–2008	11 European countries	20	25181	NA	NA	NA	NA	NA	NA	NA
Duarte (2013) [47]	2006–2011	Brazil	NA	618	195	31.55	NA	NA	NA	NA	NA
Mortz (2013) [46]	2010–2010	Denmark	NA	442	168	38.01	NA	NA	10	2.35	NA
Malinauskiene (2014) [45]	2010–2012	Sweden and Lithuania	20	642	73	11.37	NA	NA	274	42.68	NA
Simonsen (2014) [44]	2003–2011	Denmark	NA	2594	884	34.1	NA	NA	1162	44.8	NA
Frosch (2015) [43]	2009–2012	12 European countries	20	57123	NA	NA	NA	NA	NA	NA	NA
Wöhrl (2001) [42]	1997–2000	Austria	20	3407	700	20.55	NA	NA	NA	NA	NA
Fall (2015) [41]	1991–2010	Sweden	20	10599	3818	36.02	NA	NA	NA	NA	NA
Toholka (2015) [40]	2001–2010	Australia	20	5281	1865	35.32	NA	NA	1576	29.84	NA
Fortina (2015) [7]	2002–2010	11 European countries	NA	6708	2743	40.89	NA	NA	2644	39.42	NA
Mortazavi (2016) [39]	2007–2009	Iran	20	109	37	33.9	14.4 (5–18)^a^	14.4 ± 3.4	NA	NA	NA
Shi (2016) [38]	2003–2015	United States	NA	4071	NA	NA	NA	NA	NA	NA	NA
Yu (2017) [37]	2009–2014	South Korea	20	330	100	30.3	NA	40.6 ± 16.2	NA	NA	NA
Linauskiene (2017) [36]	2014–2015	Lithuania	20	297	40	13.5	NA	NA	70	23.6	NA
Ortiz Salvador (2017) [35]	2000–2015	Spain	NA	265	110	41.5	NA	11.1 ± 3.7	NA	NA	NA
Teo (2018) [34]	1984–2014	United Kingdom	NA	46250	19189	41.49	NA	NA	14732	31.86	13592
Mauro (2018) [33]	1996–2016	Italy	NA	27381	8850	32.3	NA	NA	2004	7.32	33
Sharma (2018) [32]	2010–2014	India	20	101	69	68.32	49.94 (21–78)^a^	NA	NA	NA	NA
Winayanuwattikun (2019) [31]	2007–2016	Thailand	20	2178	NA	NA	NA	41.9 ± 15.6	NA	NA	NA
Aalto‐Korte (2020) [30]	2005–2016	Finland	NA	5265	2039	38.7	NA	NA	NA	NA	NA
Felmingham (2020) [29]	1993–2017	Australia	NA	511	199	38.9	NA	NA	212	41.5	NA
Atwater (2021) [12]	2007–2016	Canada and United States	20	24246	7595	31.3	NA	NA	NA	NA	NA
Özkaya (2021) [28]	1996–2019	Turkey	NA	294	NA	NA	33 (14–67)^a^	NA	14	4.8	NA
Uter (2021) [27]	2007–2012	Germany	1.2^b^ & 20	2213	797	36	NA	NA	369	16.7	NA
Lin (2021) [26]	1978–2018	Taiwan	NA	4005	1209	30.19	NA	NA	NA	NA	NA
Seine (2021) [25]	2008–2020	New Zealand	20	2402	792	33	NA	NA	NA	NA	NA
Murphy (2021) [24]	2012–2020	New Zealand	20	820	263	32.07	NA	NA	NA	NA	NA
Boyvat (2021) [23]	2013–2019	Turkey	20	1309	545	41.6	41 (3–87)^a^	40.9 ± 16.8	66	5	20
Yilmaz (2021) [22]	1996–2017	Turkey	20	317	NA	NA	NA	NA	NA	NA	20
Andernord (2022) [21]	2010–2017	Sweden	20	21663	6700	31	NA	NA	NA	NA	NA
Wee (2022) [20]	2007–2017	Singapore	20	4903	2043	41.7	NA	40.7 ± 16.28	2529	51.6	1280
Slodownik (2022) [19]	NA	Israel	NA	301	124	41.3	NA	NA	60	19.9	NA
Uter (2022) [18]	2019–2020	13 European countries	20	22474	NA	NA	NA	NA	NA	NA	NA
Ünal (2023) [17]	2012–2022	Turkey	NA	1012	433	42.8	37.97 (18–87)^a^	NA	NA	NA	NA
Sari (2023) [16]	2011–2020	Indonesia	NA	616	196	31.82	36.5 (4–76)^a^	NA	20	3.25	NA
Katran (2024) [15]	2018–2023	Turkey	NA	383	159	41.51	NA	NA	NA	NA	NA
Boonchai (2024) [83]	2001–2021	Thailand	20	5998	1279	21.32	NA	41.9 ± 15.3	791	13.19	NA
Kim (2024) [13]	2019–2020	South Korea	NA	273	70	25.6	NA	42.6 ± 15.9	74	27.1	66
Pesqué (2025) [14]	2019–2023	Spain	NA	13368	4046	30.3	NA	NA	2426	18.1	395

*Note*: If nothing else denoted, then the vehicle is petrolatum.

Abbreviations: IQR, interquartile range; *n*, number; NA, not applicable.

^a^ Average (range).

^b^ mg/cm^2^.

### 3.2 Quantitative Assessment

#### 3.2.1 Contact Allergy to Colophonium in All Patients

**FIGURE 1:**
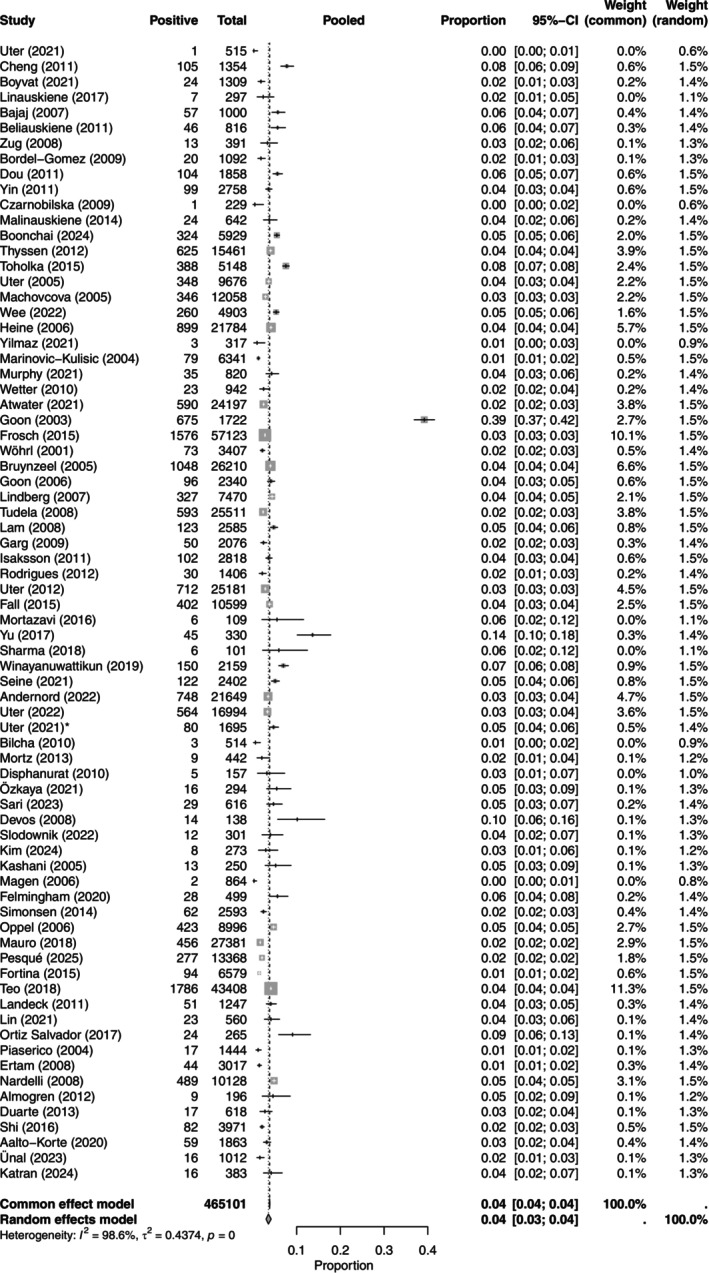
Forest plot of the prevalence of contact allergy to colophonium in dermatitis patients. Proportion meta‐analysis plot with random effects of dermatitis patients tested with colophonium. The figure displays the author, publication year, prevalence, pooled prevalence, confidence intervals and weight of each study.

The prevalence of contact allergy to colophonium was reported in 459 757 patients (*n* = 73 studies [7, 12, 13, 14, 15, 16, 17, 18, 19, 20, 21, 22, 23, 24, 25, 26, 27, 28, 29, 30, 31, 32, 33, 34, 35, 36, 37, 38, 39, 40, 41, 42, 43, 44, 45, 46, 47, 48, 49, 50, 51, 52, 53, 54, 55, 56, 57, 58, 59, 60, 61, 62, 63, 64, 65, 66, 67, 68, 69, 70, 71, 72, 73, 74, 75, 76, 77, 78, 79, 80, 81, 82, 83]) with 16 465 PPTs, yielding a pooled prevalence of 3.54% (95% CI, 3.01–4.16) and an *I* ^2^ of 98.6% (95% CI, 98.5–98.7). The clinical relevance of PPTs was 39.42% ([95% CI, 37.94–40.92], *n* = 23 studies [12, 13, 22, 23, 24, 26, 29, 35, 40, 43, 44, 46, 52, 53, 57, 60, 66, 69, 70, 72, 76, 79, 82]) (Table 2 and Figure 1).

**TABLE 2:** Proportion of positive patch tests to colophonium in all patients.

Allergen	Studies (*n*)	Patients undergoing patch testing (*n*)	Proportion of PPTs (%)	95% CI	Tau^2^ (95% CI)	*I* ^2^ (95% CI) (%)	Clinical relevance studies (*n*)	Clinical relevance (%)	95% CI
Colophonium	73	465 101	3.54	3.01; 4.16	0.44 [0.34; 0.74]	98.6% [98.5%; 98.7%]	23	39.42	37.94; 40.92

Abbreviations: CI, confidence interval; *n*, number; PPTs, positive patch tests.

#### 3.2.2 Contact Allergy to Colophonium in Patients With and Without AD

A total of seven studies [7, 20, 33, 34, 51, 61, 68] compared 31 452 patients with AD and 91 770 patients without AD. The pooled prevalence was 4.74% (95% CI, 0.29–44.77) for patients with AD and 4.87% (95% CI, 0.97–21.16) for patients without AD, with no significant difference (chi^2^‐test; *p* = 0.99) (Table 3).

**TABLE 3:** Comparison analysis of dermatitis patients with versus without AD.

Allergen	Studies (*n*)	Non‐AD			AD			
		Non‐AD patients undergoing patch testing (*n*)	Proportion of PPTs (%)	95% CI	AD patients undergoing patch testing (*n*)	Proportion of PPTs (%)	95% CI	Test for subgroup differences (random effects): Chi‐squared *p*
Colophonium	7	91 770	4.87	0.97; 21.16	31 452	4.74	0.29; 44.77	0.99

Abbreviations: AD, atopic dermatitis; CI, confidence interval; *n*, number; PPTs, positive patch tests.

#### 3.2.3 Contact Allergy to Colophonium According to Geographical Regions

Across geographical regions, the highest prevalences of contact allergy to colophonium were found in Southeast Asia (6.83% [95% CI, 3.62–12.49], *n* = 9 studies [16, 20, 31, 32, 66, 67, 69, 82, 83]), Oceania (5.69% [95% CI, 3.85–8.33], *n* = 4 studies [24, 25, 29, 40]), and East Asia (5.56% [95% CI, 3.45–8.86], *n* = 7 studies [13, 26, 37, 52, 54, 56, 62]). The lowest prevalence was found in Africa (0.68% [95% CI, 0.24–1.92], *n* = 1 study [58]). In Europe, the prevalence was 3.12% ([95% CI, 2.61–3.72], *n* = 33 studies [7, 14, 18, 21, 27, 30, 33, 34, 35, 36, 41, 42, 43, 44, 45, 46, 48, 51, 55, 57, 59, 61, 64, 65, 68, 70, 73, 74, 77, 78, 79, 80, 81]) (Table 4).

**TABLE 4:** Proportion of positive patch tests to colophonium according to geographical regions.

Europe		Middle East		North America		South America		East Asia		Southeast Asia		Oceania		Africa	
Studies (*n*)	Proportion of PPTs (95% CI) (%)	Studies (*n*)	Proportion of PPTs (95% CI) (%)	Studies (*n*)	Proportion of PPTs (95% CI) (%)	Studies (*n*)	Proportion of PPTs (95% CI) (%)	Studies (*n*)	Proportion of PPTs (95% CI) (%)	Studies (*n*)	Proportion of PPTs (95% CI) (%)	Studies (*n*)	Proportion of PPTs (95% CI) (%)	Studies (*n*)	Proportion of PPTs (95% CI) (%)
Colophonium															
33	3.12 [2.61; 3.72]	11	2.68 [1.51; 4.73]	6	2.64 [2.02; 3.46]	2	2.39 [0.46; 11.44]	7	5.56 [3.45; 8.86]	9	6.83 [3.62; 12.49]	4	5.69 [3.85; 8.33]	1	0.68 [0.24; 1.92]

Abbreviations: CI, confidence interval; *n*, number; PPTs, positive patch tests.

#### 3.2.4 Contact Allergy to Colophonium According to Study Period

**FIGURE 2:**
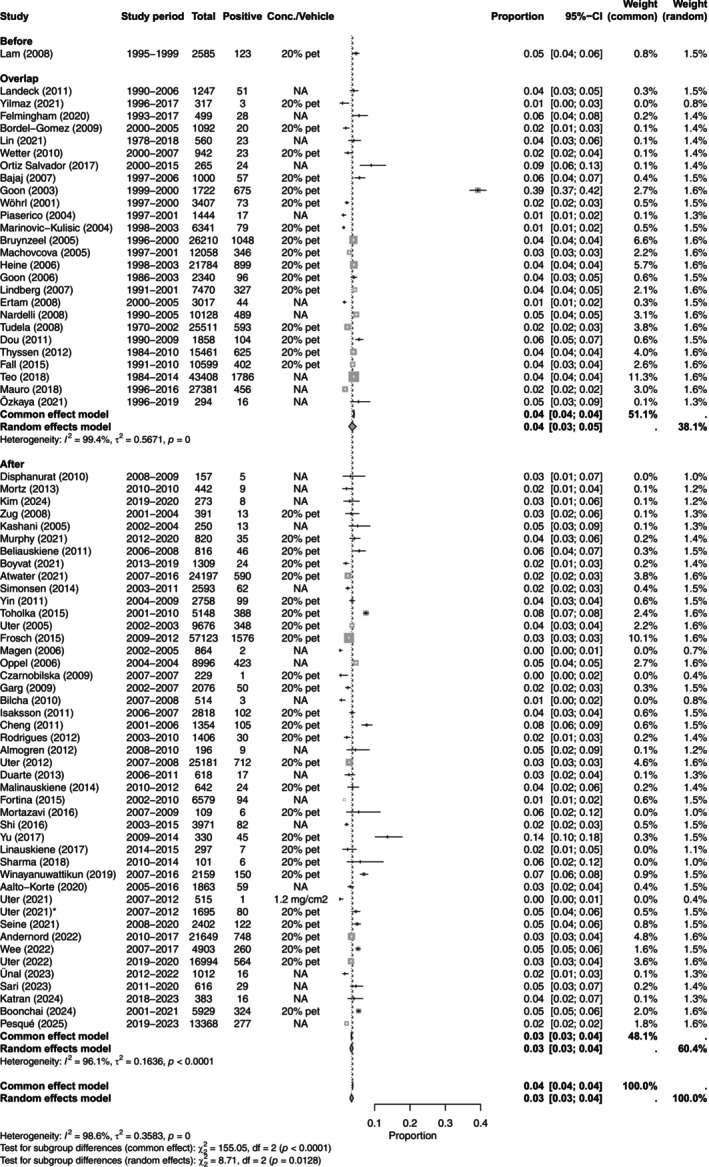
Forest plot of the prevalence of contact allergy to colophonium in dermatitis patients before, overlapping and after the year 2000. Proportion meta‐analysis plot with random effects of dermatitis patients tested with colophonium before, overlapping and after the year 2000. The figure displays the time period, author, publication year, study period, prevalence, concentration, pooled prevalence, confidence intervals and weight of each study.

One study [62] had a study period before the year 2000, with a prevalence of 4.76% (95% CI, 4.00–5.65). In total, 26 studies [22, 26, 28, 29, 33, 34, 35, 41, 42, 51, 53, 56, 60, 63, 64, 65, 66, 67, 68, 70, 71, 77, 78, 80, 81, 82] had a study period overlapping the year 2000, with a pooled prevalence of 3.45% (95% CI, 3.03–3.92).44 studies [7, 12, 13, 14, 15, 16, 17, 18, 20, 21, 23, 24, 25, 27, 30, 31, 32, 36, 37, 38, 39, 40, 43, 44, 45, 46, 47, 48, 49, 50, 52, 54, 55, 57, 58, 59, 61, 69, 72, 74, 75, 76, 79, 83] had a study period after the year 2000, with a pooled prevalence of 3.62% (95% CI, 2.71–4.80). Two studies [19, 73] did not specify their study period. The differences between the three time periods were significant (chi^2^‐test; *p* = 0.0128) (Table 5 and Figure 2).

**TABLE 5:** Proportion of positive patch tests according to study period before and after year 2000.

Allergen	Study period (year)	Studies (*n*)	Proportion of PPTs (95% CI) (%)	Test for subgroup differences (random effects): Chi‐squared *p*
Colophonium	< 2000	1	4.76 [4.00; 5.65]	0.0128
	> 2000	44	3.62 [2.71; 4.80]	
	Overlapping 2000	26	3.45 [3.03; 3.92]	

Abbreviations: CI, confidence interval; *n*, number; PPTs, positive patch tests.

#### 3.2.5 Contact Allergy to Colophonium According to Age

**FIGURE 3:**
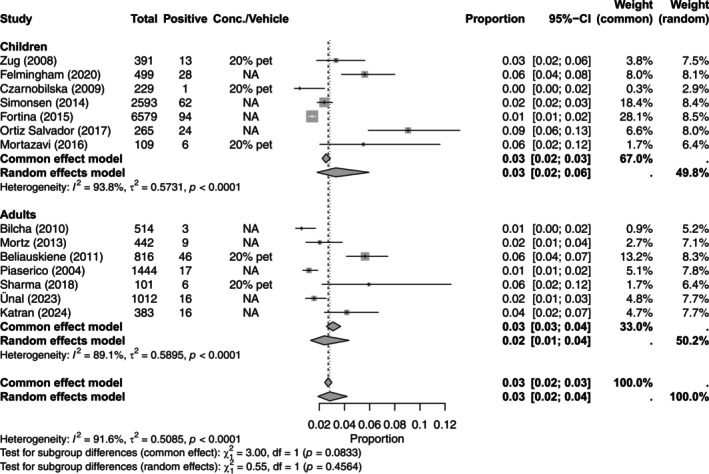
Forest plot of the prevalence of contact allergy to colophonium in dermatitis patients in children and adults. Proportion meta‐analysis plot with random effects of dermatitis patients tested with colophonium in children and adults. The figure displays the age, author, publication year, prevalence, concentration, pooled prevalence, confidence intervals and weight of each study.

The prevalence of contact allergy to colophonium was reported in seven studies each for children [7, 29, 35, 39, 44, 61, 72] and adults [15, 17, 32, 46, 57, 58, 81]. The pooled prevalence was 3.31% (95% CI, 1.82–5.96) in children and 1.31% (95% CI, 1.31–4.36) in adults. No significant difference was found between children and adults (chi^2^‐test; *p* = 0.46) (Table 6 and Figure 3).

**TABLE 6:** Proportion of positive patch tests according to age.

Allergen	Age	Studies (*n*)	Proportion of PPTs (95% CI) (%)	Test for subgroup differences (random effects): Chi‐squared *p*
Colophonium	Children	7	3.31 [1.82; 5.96]	0.46
	Adults	7	1.31 [1.31; 4.36]	

Abbreviations: CI, confidence interval; *n*, number; PPTs, positive patch tests.

#### 3.2.6 Concomitant Reactions to Colophonium

Concomitant reactions to colophonium were reported in 56 779 patients (*n* = 5 studies [12, 33, 38, 70, 73]). The proportion was 22.78% (95% CI, 20.37–25.34) for fragrance mix 1 (FM1), 14.29% (95% CI, 11.73–17.16) for fragrance mix 2 (FM2), 20.00% (95% CI, 5.73–43.66) for fragrance mix 1/2 (FM1/2), and 22.74% (95% CI, 20.34–25.27) for balsam of Peru (BOP) (Table 7).

**TABLE 7:** Total combined CR rates.

Compound 1	Compound 2	CR, %	95% CI
Colophonium	FM1	22.78	[20.37; 25.34]
	FM2	14.29	[11.73; 17.16]
	FM1/2	20.0	[5.73; 43.66]
	BOP	22.74	[20.34; 25.27]

Abbreviations: BOP, balsam of Peru; CI, confidence interval; CR, concomitant reactions; FM1, fragrance mix 1; FM1/2, fragrance mix 1 or 2; FM2, fragrance mix 2.

## 4 Discussion

This systematic review and meta‐analysis of 73 studies encompasses 459 757 dermatitis patients.

The overall prevalence of contact allergy to colophonium in dermatitis patients was 3.54%. This aligns with a previous study in patients with suspected occupational dermatitis in Finland with a prevalence of 4.6% [84] and a Swedish study on dermatitis patients with a prevalence of approximately 5% [85]. This prevalence is higher compared to other resins from the EBS such as epoxy resin (1.29%) and *p*‐*tert*‐butylphenol formaldehyde resin (PTBFR; 0.47%) [18]. The clinical relevance of colophonium was found to be 39.42%. It would be valuable to compare this result with the clinical relevance of other resins, but such studies are not available. This emphasizes the need for better registration of clinical relevance [86, 87].

We found no significant difference in the prevalence of contact allergy between patients with AD (4.74%) and without (4.87%) AD. The sub‐analysis was limited to seven studies, which restricts the generalizability of the findings. Therefore, they should be interpreted with caution. Nevertheless, this finding is consistent with other studies [88, 89].

Our sub‐analysis of age showed a numerically higher prevalence in children (3.31%) than in adults (1.31%). However, the difference was not significant and the CI was broad. These results suggest that sensitization can occur early in life from sources such as adhesive plasters, arts and crafts supplies. Further, the prevalence of contact allergy to colophonium in children in the current study is higher than that of a recent meta‐analysis on children (1.6%) [87]. However, the latter included data from 12 studies from years 2010–2024, while the current study included data from 1993 onwards.

Geographic regions showed differences in colophonium sensitization, with the highest prevalence rates found in Southeast Asia (6.83%), Oceania (5.69%) and East Asia (5.56%), and the lowest in Africa (0.68%). However, the latter was based on only one study and should be interpreted with caution. These disparities likely reflect regional differences in exposure patterns, which are shaped by local industrial practices, the use of protective equipment, the composition of consumer products and cultural habits. The higher rates in Asian regions may be linked to the use of colophonium in traditional medicines [90], cosmetics and specific industrial applications prevalent in those areas due to increased occupational exposure among workers [91, 92].

Regarding temporal trends, our findings show that the prevalence of colophonium contact allergy decreased from 4.76% in studies conducted before 2000 to 3.45% in studies conducted overlapping the year 2000. Since then, the prevalence has remained stable at 3.62%. It is important to note that the prevalence prior to 2000 is based on only one study. This stabilization contrasts with previous literature reporting a continuous decline [33] and may indicate that the benefits of global product reformulation have plateaued. This stabilization is concerning compared to the successful regulatory control of other allergens, such as rubber accelerators [93] and methylisothiazolinone [41, 94].

Historically, colophonium was used in bandages and medical adhesives [95], until modern bandages switched to acrylate‐based adhesives [96]. Nevertheless, colophonium remains a prevalent allergen in modern medical adhesives [97]. A notable outbreak of severe foot dermatitis in military conscripts was traced to colophonium in medical adhesive tape used to prevent blisters, with 61% of affected individuals testing positive [98]. Additionally, medical devices such as continuous glucose monitors and insulin pumps are a significant source of exposure, with colophonium identified as a key allergen in the adhesives of insulin devices [99]. The persistent and stable prevalence of contact allergy to colophonium suggests a lack of intervention. This is likely due to its widespread and often undeclared presence in countless products, making it an intractable public health target [84].

Furthermore, colophonium co‐sensitizes with fragrance allergens, including FM1 (22.78%), FM2 (14.29%) and BOP (22.74%) (Table 7). This strong association suggests frequent co‐exposure to these allergens in products such as cosmetics and topical medications. Therefore, patients with contact allergy to colophonium should be considered at high risk for fragrance allergies. However, studies reporting rates of co‐sensitization are scarce, and future studies should attempt to report this.

The primary strength of this meta‐analysis is the inclusion of 73 studies comprising 459 757 patients, thus providing a generalizable estimate of the prevalence of contact allergy to colophonium. Further, the extensive sub‐analyses provided valuable insights into the epidemiology of contact allergy to colophonium. However, the findings are subject to limitations, including significant statistical heterogeneity among studies, reflecting true differences in populations and testing methods. Furthermore, the validity of our results is constrained by the inherent diagnostic challenges of patch testing for colophonium [99].

## 5 Gaps in Knowledge

Significant knowledge gaps exist regarding colophonium, primarily due to inadequate labelling and disclosure of its presence in occupational and consumer products, including adhesives in medical devices [3, 84, 100]. This lack of transparency hinders clinicians' ability to accurately assess exposure and identify sensitizing agents, even in ‘hypoallergenic’ products [101, 102].

Further research is needed to fully understand the complex composition of colophonium and its derivatives, as well as the mechanisms of cross‐reactivity with other allergens [103].

## 6 Conclusion

This meta‐analysis found a pooled prevalence of contact allergy to colophonium of 3.54% (95% CI, 3.01–4.16) across 73 studies among dermatitis patients. This confirms that contact allergy to colophonium is a common and persistent problem among dermatitis patients worldwide. The stable prevalence in recent decades, coupled with its hidden presence in consumer, medical, and occupational products, highlights the necessity of collective primary prevention efforts and improved labelling to reduce the burden of colophonium allergy.

## Author Contributions

**Daniel Isufi:** investigation, writing – review and editing, validation, resources, methodology, data curation. **Kian Karimian:** conceptualization, investigation, writing – original draft, methodology, validation, writing – review and editing, project administration, supervision. **Mikkel Bak Jensen:** software, supervision, formal analysis, writing – review and editing, visualization, validation, methodology, conceptualization. **Jeanne Duus Johansen:** conceptualization, validation, writing – review and editing, project administration, supervision. **Jakob Ferløv Baselius Schwensen:** conceptualization, validation, supervision, project administration, writing – review and editing.

## Funding

The Danish Environmental Protection Agency under the Ministry of Environment of Denmark.

## Conflicts of Interest

Dr. Schwensen has previously served as a speaker and investigator for Galderma and Sanofi‐Aventis. The other authors declare no conflicts of interest.

## Supporting information


**Table S1:** Search strings for databases.
**Table S2:** Appraisal tool for Cross‐Sectional Studies (AXIS) assessment of included studies.
**Figure S1:** The Preferred Reporting Items for Systematic Reviews and Meta‐analyses (PRISMA) flowchart.
**Figure S2:** Funnel plot of contact allergy to colophonium in all patients.

**Data S1:** PRISMA flow colophonium.

**Data S2:** PRISMA checklist Colophonium_1.

**Data S3:** PRISMA checklist Colophonium_2.

## Data Availability

The data that support the findings of this study are available from the corresponding author upon reasonable request.
