# Supplementary material for: Prevalence of Contact Allergy to Colophonium in Dermatitis Patients: A Systematic Review and Meta‐Analysis
Source: Contact Dermatitis. 2026 Apr 5;95(1):1–16. doi: 10.1111/cod.70153 (PMC13238359; doi:10.1111/cod.70153)
Supplement: Supplementary file 1 — Table S1: Search strings for databases. Table S2: Appraisal tool for Cross‐Sectional Studies (AXIS) assessment of included studies. Figure S1: The Preferred Reporting Items for Systematic Reviews and Meta‐analyses (PRISMA) flowchart. Figure S2: Funnel plot of contact allergy to colophonium in all patients. [file COD-95-1-s001.docx]

**Supplementary Material**

[Supplementary Tables 2](#_Toc209190138)

[Supplementary Table 1: Search strings for databases 2](#_Toc209190139)

[Supplementary Table 2: Appraisal tool for Cross-Sectional Studies (AXIS) assessment of included studies 3](#_Toc209190140)

[Supplementary Figures 7](#_Toc209190141)

[Supplementary Figure 1: The Preferred Reporting Items for Systematic Reviews and Meta-analyses (PRISMA) flowchart 7](#_Toc209190142)

[Supplementary Figure 2: Funnel plot of contact allergy to colophonium in all patients 8](#_Toc209190143)

[References 9](#_Toc209190144)

# **Supplementary Tables**

| Supplementary Table 1: Search strings for databases |
| --- |
| (”colophonium” OR ”colophony” OR ”rosin” OR ”gum rosin” OR ”wood rosin” OR ”tall oil rosin” OR ”abietic acid” OR ”abietyl alcohol”)  AND  (“allergic contact dermatitis” OR “contact allergy” OR “contact dermatitis” OR “allergic reaction” OR “hypersensitivity” OR “contact sensitization” OR “contact sensitivity”) |

| Supplementary Table 2: Appraisal tool for Cross-Sectional Studies (AXIS) assessment of included studies | | | | | | | | | | | | | | | | | | | | |
| --- | --- | --- | --- | --- | --- | --- | --- | --- | --- | --- | --- | --- | --- | --- | --- | --- | --- | --- | --- | --- |
|  | **Introduction** | **Methods** | | | | | | | | | | **Results** | | | | | **Discussion** | | **Other** | |
| **Reference** | Q1 | Q2 | Q3 | Q4 | Q5 | Q6 | Q7 | Q8 | Q9 | Q10 | Q11 | Q12 | Q13 | Q14 | Q15 | Q16 | Q17 | Q18 | Q19 | Q20 |
| Goon (2003)^83^ | 1 | 1 | 1 | 1 | 1 | 1 | 3 | 1 | 1 | 1 | 1 | 1 | 2 | 3 | 1 | 1 | 1 | 2 | 3 | 3 |
| Piaserico (2004)^82^ | 1 | 1 | 1 | 1 | 1 | 1 | 3 | 1 | 1 | 1 | 1 | 1 | 2 | 3 | 1 | 1 | 1 | 2 | 3 | 3 |
| Marinovic-Kulisic (2004)^81^ | 1 | 1 | 1 | 1 | 1 | 1 | 3 | 1 | 1 | 1 | 1 | 1 | 2 | 3 | 1 | 1 | 1 | 2 | 3 | 3 |
| Uter (2005)^80^ | 1 | 1 | 1 | 1 | 1 | 1 | 3 | 1 | 1 | 1 | 1 | 1 | 2 | 3 | 1 | 1 | 1 | 2 | 3 | 3 |
| Bruynzeel (2005)^79^ | 1 | 1 | 1 | 1 | 1 | 1 | 3 | 1 | 1 | 1 | 1 | 1 | 2 | 3 | 1 | 1 | 1 | 2 | 3 | 3 |
| Machovcova (2005)^78^ | 1 | 1 | 1 | 1 | 1 | 1 | 3 | 1 | 1 | 1 | 1 | 1 | 2 | 3 | 1 | 1 | 1 | 2 | 3 | 3 |
| Kashani (2005)^77^ | 1 | 1 | 1 | 1 | 1 | 1 | 3 | 1 | 1 | 1 | 1 | 1 | 2 | 3 | 1 | 1 | 1 | 2 | 3 | 3 |
| Magen (2006)^76^ | 1 | 1 | 1 | 1 | 1 | 1 | 3 | 1 | 1 | 1 | 1 | 1 | 2 | 3 | 1 | 1 | 1 | 1 | 3 | 3 |
| Oppel (2006)^75^ | 1 | 1 | 1 | 1 | 1 | 1 | 3 | 1 | 1 | 1 | 1 | 1 | 2 | 3 | 1 | 1 | 1 | 2 | 2 | 3 |
| Devos (2008)^74^ | 1 | 1 | 1 | 1 | 1 | 1 | 3 | 1 | 1 | 1 | 1 | 1 | 2 | 3 | 1 | 1 | 1 | 2 | 3 | 3 |
| Zug (2008)^73^ | 1 | 1 | 1 | 1 | 1 | 1 | 3 | 1 | 1 | 1 | 1 | 1 | 2 | 3 | 1 | 1 | 1 | 1 | 3 | 1 |
| Ertam (2008)^72^ | 1 | 1 | 1 | 1 | 1 | 1 | 3 | 1 | 1 | 1 | 1 | 1 | 2 | 3 | 1 | 1 | 1 | 2 | 3 | 3 |
| Bordel-Gomez (2009)^71^ | 1 | 1 | 1 | 1 | 1 | 1 | 3 | 1 | 1 | 1 | 1 | 1 | 2 | 3 | 1 | 1 | 1 | 2 | 2 | 1 |
| Disphanurat (2010)^70^ | 1 | 1 | 1 | 1 | 1 | 1 | 3 | 1 | 1 | 1 | 1 | 1 | 2 | 3 | 1 | 1 | 1 | 1 | 3 | 3 |
| Heine (2006)^69^ | 1 | 1 | 1 | 1 | 1 | 1 | 3 | 1 | 1 | 1 | 1 | 1 | 2 | 3 | 1 | 1 | 1 | 2 | 3 | 3 |
| Goon (2006)^68^ | 1 | 1 | 1 | 1 | 1 | 1 | 3 | 1 | 1 | 1 | 1 | 1 | 2 | 3 | 1 | 1 | 1 | 1 | 3 | 3 |
| Bajaj (2007)^67^ | 1 | 1 | 1 | 1 | 1 | 1 | 3 | 1 | 1 | 1 | 1 | 1 | 2 | 3 | 1 | 1 | 1 | 2 | 3 | 3 |
| Lindberg (2007)^66^ | 1 | 1 | 1 | 1 | 1 | 1 | 3 | 1 | 1 | 1 | 1 | 1 | 2 | 3 | 1 | 1 | 1 | 1 | 3 | 3 |
| Nardelli (2008)^65^ | 1 | 1 | 1 | 1 | 1 | 1 | 3 | 1 | 1 | 1 | 1 | 1 | 2 | 3 | 1 | 1 | 1 | 2 | 3 | 3 |
| Tudela (2008)^64^ | 1 | 1 | 1 | 1 | 1 | 1 | 3 | 1 | 1 | 1 | 1 | 2 | 2 | 3 | 1 | 1 | 1 | 2 | 3 | 3 |
| Lam (2008)^63^ | 1 | 1 | 1 | 1 | 1 | 1 | 3 | 1 | 1 | 1 | 1 | 1 | 2 | 3 | 1 | 1 | 1 | 1 | 3 | 3 |
| Czarnobilska (2009)^62^ | 1 | 1 | 1 | 1 | 1 | 1 | 3 | 1 | 1 | 1 | 1 | 2 | 2 | 3 | 1 | 1 | 1 | 2 | 2 | 3 |
| Wetter (2010)^61^ | 1 | 1 | 1 | 1 | 1 | 1 | 3 | 1 | 1 | 1 | 1 | 1 | 2 | 3 | 1 | 1 | 1 | 1 | 2 | 1 |
| Garg (2009)^60^ | 1 | 1 | 1 | 1 | 1 | 1 | 3 | 1 | 1 | 1 | 1 | 1 | 2 | 3 | 1 | 1 | 1 | 2 | 3 | 3 |
| Bilcha (2010)^59^ | 1 | 1 | 1 | 1 | 1 | 1 | 3 | 1 | 1 | 1 | 1 | 1 | 2 | 3 | 1 | 1 | 1 | 2 | 2 | 3 |
| Beliauskiene (2011)^58^ | 1 | 1 | 1 | 1 | 1 | 1 | 3 | 1 | 1 | 1 | 1 | 1 | 2 | 3 | 1 | 1 | 1 | 2 | 2 | 1 |
| Dou (2011)^57^ | 1 | 1 | 1 | 1 | 1 | 1 | 3 | 1 | 1 | 1 | 1 | 1 | 2 | 3 | 1 | 1 | 1 | 1 | 2 | 3 |
| Isaksson (2011)^56^ | 1 | 1 | 1 | 1 | 1 | 1 | 3 | 1 | 1 | 1 | 1 | 1 | 2 | 3 | 1 | 1 | 1 | 2 | 3 | 3 |
| Cheng (2011)^55^ | 1 | 1 | 1 | 1 | 1 | 1 | 3 | 1 | 1 | 1 | 1 | 1 | 2 | 3 | 1 | 1 | 1 | 2 | 2 | 1 |
| Landeck (2011)^54^ | 1 | 1 | 1 | 1 | 1 | 1 | 3 | 1 | 1 | 1 | 1 | 1 | 2 | 3 | 1 | 1 | 1 | 2 | 2 | 1 |
| Yin (2011)^53^ | 1 | 1 | 1 | 1 | 1 | 1 | 3 | 1 | 1 | 1 | 1 | 1 | 2 | 3 | 1 | 1 | 1 | 1 | 2 | 3 |
| Thyssen (2012)^52^ | 1 | 1 | 1 | 1 | 1 | 1 | 3 | 1 | 1 | 1 | 1 | 1 | 2 | 3 | 1 | 1 | 1 | 1 | 2 | 3 |
| Rodrigues (2012)^51^ | 1 | 1 | 1 | 1 | 1 | 1 | 3 | 1 | 1 | 1 | 1 | 1 | 2 | 3 | 1 | 1 | 1 | 1 | 2 | 3 |
| Almogren (2012)^50^ | 1 | 1 | 1 | 1 | 1 | 1 | 3 | 1 | 1 | 1 | 1 | 2 | 2 | 3 | 1 | 1 | 1 | 2 | 3 | 3 |
| Uter (2012)^49^ | 1 | 1 | 1 | 1 | 1 | 1 | 3 | 1 | 1 | 1 | 1 | 1 | 2 | 3 | 1 | 1 | 1 | 2 | 3 | 3 |
| Duarte (2013)^48^ | 1 | 1 | 1 | 1 | 1 | 1 | 3 | 1 | 1 | 1 | 1 | 1 | 2 | 3 | 1 | 1 | 1 | 2 | 2 | 3 |
| Mortz (2013)^47^ | 1 | 1 | 1 | 1 | 1 | 1 | 3 | 1 | 1 | 1 | 1 | 1 | 2 | 3 | 1 | 1 | 1 | 2 | 3 | 1 |
| Malinauskiene (2014)^46^ | 1 | 1 | 1 | 1 | 1 | 1 | 3 | 1 | 1 | 1 | 1 | 1 | 2 | 3 | 1 | 1 | 1 | 1 | 2 | 3 |
| Simonsen (2014)^45^ | 1 | 1 | 1 | 1 | 1 | 1 | 3 | 1 | 1 | 1 | 1 | 1 | 2 | 3 | 1 | 1 | 1 | 1 | 2 | 3 |
| Frosch (2015)^44^ | 1 | 1 | 1 | 1 | 1 | 1 | 3 | 1 | 1 | 1 | 1 | 1 | 2 | 3 | 1 | 1 | 1 | 2 | 3 | 3 |
| Wöhrl (2001)^43^ | 1 | 1 | 1 | 1 | 1 | 1 | 3 | 1 | 1 | 1 | 1 | 1 | 2 | 3 | 1 | 1 | 1 | 2 | 2 | 3 |
| Fall (2015)^42^ | 1 | 1 | 1 | 1 | 1 | 1 | 3 | 1 | 1 | 1 | 1 | 1 | 2 | 3 | 1 | 1 | 1 | 1 | 2 | 1 |
| Toholka (2015)^41^ | 1 | 1 | 1 | 1 | 1 | 1 | 3 | 1 | 1 | 1 | 1 | 1 | 2 | 3 | 1 | 1 | 1 | 1 | 2 | 3 |
| Fortina (2015)^40^ | 1 | 1 | 1 | 1 | 1 | 1 | 3 | 1 | 1 | 1 | 1 | 1 | 2 | 3 | 1 | 1 | 1 | 1 | 3 | 3 |
| Mortazavi (2016)^39^ | 1 | 1 | 1 | 1 | 1 | 1 | 3 | 1 | 1 | 1 | 1 | 1 | 2 | 3 | 1 | 1 | 1 | 1 | 3 | 1 |
| Shi (2016)^38^ | 1 | 1 | 1 | 1 | 1 | 1 | 3 | 1 | 1 | 1 | 1 | 1 | 2 | 3 | 1 | 1 | 1 | 1 | 2 | 3 |
| Yu (2017)^37^ | 1 | 1 | 1 | 1 | 1 | 1 | 3 | 1 | 1 | 1 | 1 | 1 | 2 | 3 | 1 | 1 | 1 | 1 | 2 | 1 |
| Linauskiene (2017)^36^ | 1 | 1 | 1 | 1 | 1 | 1 | 3 | 1 | 1 | 1 | 1 | 1 | 2 | 3 | 1 | 1 | 1 | 1 | 2 | 3 |
| Ortiz Salvador (2017)^35^ | 1 | 1 | 1 | 1 | 1 | 1 | 3 | 1 | 1 | 1 | 1 | 1 | 2 | 3 | 1 | 1 | 1 | 2 | 2 | 3 |
| Teo (2018)^34^ | 1 | 1 | 1 | 1 | 1 | 1 | 3 | 1 | 1 | 1 | 1 | 1 | 2 | 3 | 1 | 1 | 1 | 2 | 2 | 3 |
| Mauro (2018)^33^ | 1 | 1 | 1 | 1 | 1 | 1 | 3 | 1 | 1 | 1 | 1 | 1 | 2 | 3 | 1 | 1 | 1 | 1 | 2 | 3 |
| Sharma (2018)^32^ | 1 | 1 | 1 | 1 | 1 | 1 | 3 | 1 | 1 | 1 | 1 | 1 | 2 | 3 | 1 | 1 | 1 | 1 | 2 | 3 |
| Winayanuwattikun (2019)^31^ | 1 | 1 | 1 | 1 | 1 | 1 | 3 | 1 | 1 | 1 | 1 | 1 | 2 | 3 | 1 | 1 | 1 | 2 | 2 | 1 |
| Aalto-Korte (2020)^30^ | 1 | 1 | 1 | 1 | 1 | 1 | 3 | 1 | 1 | 1 | 1 | 1 | 2 | 3 | 1 | 1 | 1 | 1 | 2 | 3 |
| Felmingham (2020)^29^ | 1 | 1 | 1 | 1 | 1 | 1 | 3 | 1 | 1 | 1 | 1 | 1 | 2 | 3 | 1 | 1 | 1 | 1 | 3 | 1 |
| Atwater (2021)^12^ | 1 | 1 | 1 | 1 | 1 | 1 | 3 | 1 | 1 | 1 | 1 | 1 | 2 | 3 | 1 | 1 | 1 | 1 | 2 | 1 |
| Özkaya (2021)^28^ | 1 | 1 | 1 | 1 | 1 | 1 | 3 | 1 | 1 | 1 | 1 | 1 | 2 | 3 | 1 | 1 | 1 | 1 | 2 | 1 |
| Uter (2021)^27^ | 1 | 1 | 1 | 1 | 1 | 1 | 3 | 1 | 1 | 1 | 1 | 1 | 2 | 3 | 1 | 1 | 1 | 2 | 3 | 3 |
| Lin (2021)^26^ | 1 | 1 | 1 | 1 | 1 | 1 | 3 | 1 | 1 | 1 | 1 | 1 | 2 | 3 | 1 | 1 | 1 | 1 | 2 | 1 |
| Seine (2021)^25^ | 1 | 1 | 1 | 1 | 1 | 1 | 3 | 1 | 1 | 1 | 1 | 2 | 2 | 3 | 1 | 1 | 1 | 1 | 3 | 1 |
| Murphy (2021)^24^ | 1 | 1 | 1 | 1 | 1 | 1 | 3 | 1 | 1 | 1 | 1 | 1 | 2 | 3 | 1 | 1 | 1 | 1 | 3 | 1 |
| Boyvat (2021)^23^ | 1 | 1 | 1 | 1 | 1 | 1 | 3 | 1 | 1 | 1 | 1 | 1 | 2 | 3 | 1 | 1 | 1 | 2 | 2 | 1 |
| Yilmaz (2021)^22^ | 1 | 1 | 1 | 1 | 1 | 1 | 3 | 1 | 1 | 1 | 1 | 2 | 2 | 3 | 1 | 1 | 1 | 1 | 2 | 1 |
| Andernord (2022)^21^ | 1 | 1 | 1 | 1 | 1 | 1 | 3 | 1 | 1 | 1 | 1 | 1 | 2 | 3 | 1 | 1 | 1 | 1 | 3 | 1 |
| Wee (2022)^20^ | 1 | 1 | 1 | 1 | 1 | 1 | 3 | 1 | 1 | 1 | 1 | 1 | 2 | 3 | 1 | 1 | 1 | 1 | 2 | 1 |
| Slodownik (2022)^19^ | 1 | 1 | 1 | 1 | 1 | 1 | 3 | 1 | 1 | 1 | 1 | 2 | 2 | 3 | 1 | 1 | 1 | 2 | 2 | 1 |
| Uter (2022)^18^ | 1 | 1 | 1 | 1 | 1 | 1 | 3 | 1 | 1 | 1 | 1 | 1 | 2 | 3 | 1 | 1 | 1 | 2 | 3 | 1 |
| Ünal (2023)^17^ | 1 | 1 | 1 | 1 | 1 | 1 | 3 | 1 | 1 | 1 | 1 | 1 | 2 | 3 | 1 | 1 | 1 | 1 | 2 | 1 |
| Sari (2023)^16^ | 1 | 1 | 1 | 1 | 1 | 1 | 3 | 1 | 1 | 1 | 1 | 2 | 2 | 3 | 1 | 1 | 1 | 2 | 3 | 3 |
| Katran (2024)^15^ | 1 | 1 | 1 | 1 | 1 | 1 | 3 | 1 | 1 | 1 | 1 | 1 | 2 | 3 | 1 | 1 | 1 | 1 | 2 | 1 |
| Boonchai (2024)^84^ | 1 | 1 | 1 | 1 | 1 | 1 | 3 | 1 | 1 | 1 | 1 | 1 | 2 | 3 | 1 | 1 | 1 | 1 | 2 | 1 |
| Kim (2024)^13^ | 1 | 1 | 1 | 1 | 1 | 1 | 3 | 1 | 1 | 1 | 1 | 1 | 2 | 3 | 1 | 1 | 1 | 1 | 3 | 1 |
| Pesqué (2025)^14^ | 1 | 1 | 1 | 1 | 1 | 1 | 3 | 1 | 1 | 1 | 1 | 1 | 2 | 3 | 1 | 1 | 1 | 1 | 3 | 1 |

# **Supplementary Figures**

## Supplementary Figure 1: The Preferred Reporting Items for Systematic Reviews and Meta-analyses (PRISMA) flowchart


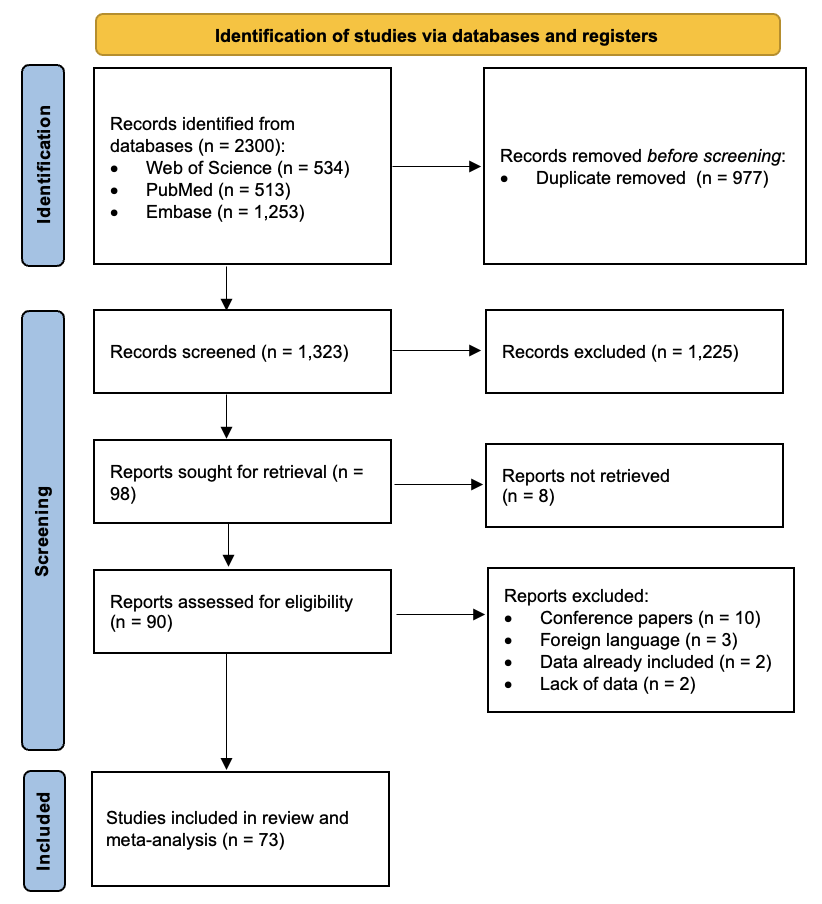


## Supplementary Figure 2: Funnel plot of contact allergy to colophonium in all patients


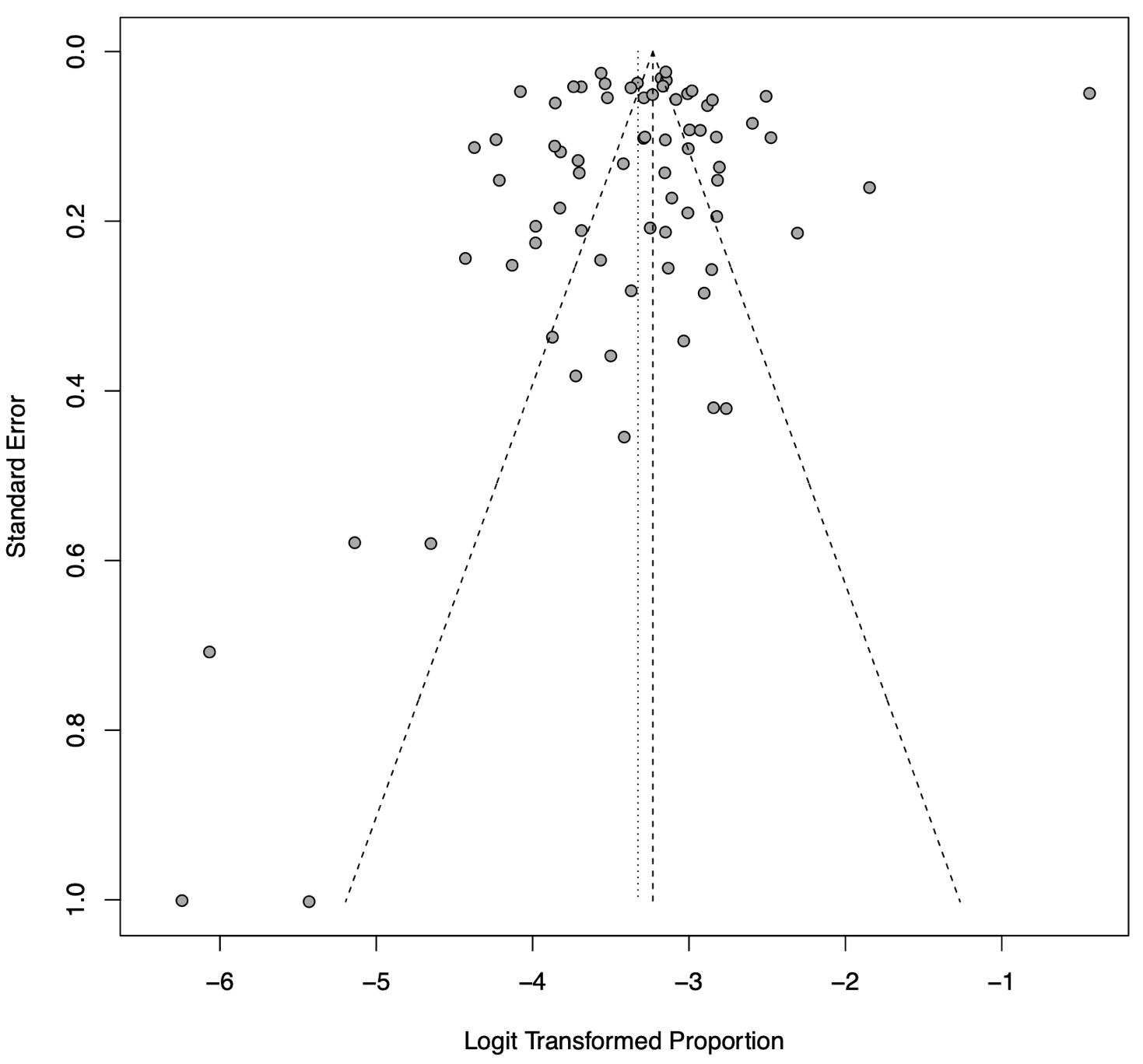


# **References**

1. Downs AMR, Sansom JE. Colophony allergy: A review. *Contact Dermatitis*. 1999;41(6):305-310. doi:10.1111/J.1600-0536.1999.TB06178.X

2. Karlberg AT, Hagvall L. Colophony: Rosin in unmodified and modified form. In: *Kanerva’s Occupational Dermatology*. 2019. doi:10.1007/978-3-319-68617-2_41

3. Karlberg AT, Albadr MH, Nilsson U. Tracing colophonium in consumer products. *Contact Dermatitis*. 2021;85(6). doi:10.1111/cod.13944

4. Maibach HI. Easier patch testing with TRUE Test. *J Am Acad Dermatol*. 1989;20(3). doi:10.1016/S0190-9622(89)70056-6

5. Wilkinson SM, Gonçalo M, Aerts O, et al. The European baseline series and recommended additions: 2023. *Contact Dermatitis*. 2023;88(2):87-92. doi:10.1111/COD.14255

6. Uter W, Amario-Hita JC, Balato A, et al. European Surveillance System on Contact Allergies (ESSCA): results with the European baseline series, 2013/14. *Journal of the European Academy of Dermatology and Venereology*. 2017;31(9):1516-1525. doi:10.1111/JDV.14423

7. Belloni Fortina A, Cooper SM, Spiewak R, Fontana E, Schnuch A, Uter W. Patch test results in children and adolescents across Europe. Analysis of the ESSCA Network 2002–2010. *Pediatric Allergy and Immunology*. 2015;26(5):446-455. doi:10.1111/PAI.12397

8. Pesonen M, Jolanki R, Larese Filon F, et al. Patch test results of the European baseline series among patients with occupational contact dermatitis across Europe – analyses of the European Surveillance System on Contact Allergy network, 2002–2010. *Contact Dermatitis*. 2015;72(3):154-163. doi:10.1111/COD.12333

9. Moher D, Liberati A, Tetzlaff J, Altman DG. Preferred reporting items for systematic reviews and meta-analyses: The PRISMA statement. *BMJ (Online)*. 2009;339(7716). doi:10.1136/bmj.b2535

10. Ouzzani M, Hammady H, Fedorowicz Z, Elmagarmid A. Rayyan-a web and mobile app for systematic reviews. *Syst Rev*. 2016;5(1):1-10. doi:10.1186/S13643-016-0384-4/FIGURES/6

11. Downes MJ, Brennan ML, Williams HC, Dean RS. Development of a critical appraisal tool to assess the quality of cross-sectional studies (AXIS). *BMJ Open*. 2016;6(12):e011458. doi:10.1136/BMJOPEN-2016-011458

12. Atwater AR, Ward JM, Liu B, et al. Fragrance- and Botanical-Related Allergy and Associated Concomitant Reactions: A Retrospective Analysis of the North American Contact Dermatitis Group Data 2007-2016. *Dermatitis*. 2021;32(1):42-52. doi:10.1097/DER.0000000000000661

13. 의과대학고신대학교, 해운대백병원 내과인제대학교, 의과대학 내과고신대학교, et al. Analysis of patch test results in patients with suspected contact dermatitis in the Busan area. *Allergy, Asthma & Respiratory Disease*. 2024;12(2):85-92. doi:10.4168/AARD.2024.12.2.85

14. Pesqué D, Planella-Fontanillas N, Borrego L, et al. Patch test results to the Spanish baseline patch test series according to age groups: A multicentric prospective study from 2019 to 2023. *Contact Dermatitis*. 2024;92(2):120. doi:10.1111/COD.14702

15. Katran ZY, Bulut İ. Results of patch testing to the European Baseline Series in adult patients in Turkey: a five-year experience at a tertiary reference center. *Alergologia Polska - Polish Journal of Allergology*. 2024;11(1). doi:10.5114/pja.2024.135510

16. Characteristics of patch test patients in Yogyakarta, Indonesia, 2011-2020 Study Period. *Journal of Pakistan Association of Dermatologists*.

17. Ünal A. Analysis of patch testing results in patients with contact dermatitis in Istanbul, Turkey, from 2012 to 2022. *J Cosmet Dermatol*. 2023;22(10):2831-2838. doi:10.1111/JOCD.15791

18. Uter W, Wilkinson SM, Aerts O, et al. Patch test results with the European baseline series, 2019/20—Joint European results of the ESSCA and the EBS working groups of the ESCD, and the GEIDAC. *Contact Dermatitis*. 2022;87(4):343-355. doi:10.1111/COD.14170

19. Slodownik D, Rabah SM, Levi A, et al. The relationship between atopy and allergic contact dermatitis in Israeli patients. *Advances in Dermatology and Allergology/Postȩpy Dermatologii i Alergologii*. 2022;39(1):159. doi:10.5114/ADA.2022.113606

20. Wee C, Tan CH, Zhao X, Yew YW, Goon A. Pattern of contact sensitization in patients with and without atopic dermatitis in an Asian dermatology center. *Contact Dermatitis*. 2022;86(5):398-403. doi:10.1111/COD.14068

21. Andernord D, Bruze M, Bryngelsson IL, et al. Contact allergy to haptens in the Swedish baseline series: Results from the Swedish Patch Test Register (2010 to 2017). *Contact Dermatitis*. 2022;86(3):175-188. doi:10.1111/COD.13996

22. Yılmaz Z, Özkaya E, Mahallesi Ç, Yıl B 75, Hastanesi D, Klini D. Patch test results in terms of the recently recommended allergens in children and adolescents: A retrospective cohort study over 22 years from Turkey. *Contact Dermatitis*. 2021;85(2):198-210. doi:10.1111/COD.13842

23. Boyvat A, Kalay Yildizhan I. Patch test results of the European baseline series among 1309 patients in Turkey between 2013 and 2019. *Contact Dermatitis*. 2021;84(1):15-23. doi:10.1111/COD.13653

24. Murphy VL, Patel C, Lamb SR, Cheng HS. Patch testing to plants: sensitisation associated with exposure to plants, essential oils and botanicals in cosmetics. 2021;134:1536. Accessed September 16, 2025. www.nzma.org.nz/journal

25. Seine AJ, Baird EA, Chan L, et al. A baseline patch test series for New Zealand. *Australasian Journal of Dermatology*. 2021;62(4):489-495. doi:10.1111/AJD.13673

26. Lin PH, Tseng YH, Chu CY. Changing trends of contact allergens: A 40-year retrospective study from a referral centre in northern Taiwan. *Contact Dermatitis*. 2021;85(1):39-45. doi:10.1111/COD.13795

27. Uter W, Zetzmann A, Ofenloch R, et al. Prevalence of contact allergies in the population compared to a tertiary referral patch test clinic in Jena/Germany. *Contact Dermatitis*. 2021;85(5):563-571. doi:10.1111/COD.13923

28. Özkaya E, Elinç Aslan MS. Occupational allergic contact dermatitis: A 24-year, retrospective cohort study from Turkey. *Contact Dermatitis*. 2021;85(5):503-513. doi:10.1111/COD.13938

29. Felmingham C, Davenport R, Bala H, Palmer A, Nixon R. Allergic contact dermatitis in children and proposal for an Australian Paediatric Baseline Series. *Australasian Journal of Dermatology*. 2020;61(1):33-38. doi:10.1111/AJD.13169

30. Aalto-Korte K, Koskela K, Pesonen M. 12-year data on dermatologic cases in the Finnish Register of Occupational Diseases I: Distribution of different diagnoses and main causes of allergic contact dermatitis. *Contact Dermatitis*. 2020;82(6):337-342. doi:10.1111/COD.13488

31. Winayanuwattikun W, Boonchai W. Factors associated with multiple contact allergies in Thai dermatitis patients: A 10-year retrospective study. *Contact Dermatitis*. 2019;80(5):279-285. doi:10.1111/COD.13189

32. Sharma VK, Bhari N, Wadhwani AR, Bhatia R. Photo-patch and patch tests in patients with dermatitis over the photo-exposed areas: A study of 101 cases from a tertiary care centre in India. *Australasian Journal of Dermatology*. 2018;59(1):e1-e5. doi:10.1111/AJD.12504

33. Mauro M, Fortina AB, Corradin T, Marino A, Bovenzi M, Filon FL. Sensitization to, and allergic contact dermatitis caused by, colophonium in north-eastern Italy in 1996 to 2016 with a focus on occupational exposures. *Contact Dermatitis*. 2018;79(5):303-309. doi:10.1111/COD.13089

34. Teo Y, McFadden JP, White IR, Lynch M, Banerjee P. Allergic contact dermatitis in atopic individuals: Results of a 30-year retrospective study. *Contact Dermatitis*. 2019;81(6):409-416. doi:10.1111/COD.13363

35. Ortiz Salvador JM, Esteve Martínez A, Subiabre Ferrer D, Victoria Martínez AM, de la Cuadra Oyanguren J, Zaragoza Ninet V. Pediatric Allergic Contact Dermatitis: Clinical and Epidemiological Study in a Tertiary Hospital. *Actas Dermo-Sifiliográficas (English Edition)*. 2017;108(6):571-578. doi:10.1016/J.ADENGL.2017.05.012

36. Linauskienė K, Malinauskienė L, Blažienė A. Time trends of contact allergy to the European baseline series in Lithuania. *Contact Dermatitis*. 2017;76(6):350-356. doi:10.1111/COD.12726

37. Yu DS, Kim HJ, Park YG, Bae JM, Kim JW, Lee YB. Patch-test results using Korean standard series: a 5-year retrospective review. *Journal of Dermatological Treatment*. 2017;28(3):258-262. doi:10.1080/09546634.2016.1219015

38. Shi Y, Nedorost S, Scheman L, Scheman A. Propolis, Colophony, and Fragrance Cross-Reactivity and Allergic Contact Dermatitis. *Dermatitis*. 2016;27(3):123-126. doi:10.1097/DER.0000000000000186

39. Mortazavi H, Ehsani A, Sajjadi SS, Aghazadeh N, Arian E. Patch testing in Iranian children with allergic contact dermatitis. *BMC Dermatol*. 2016;16(1):1-6. doi:10.1186/S12895-016-0047-0/TABLES/5

40. Belloni Fortina A, Cooper SM, Spiewak R, Fontana E, Schnuch A, Uter W. Patch test results in children and adolescents across Europe. Analysis of the ESSCA Network 2002–2010. *Pediatric Allergy and Immunology*. 2015;26(5):446-455. doi:10.1111/PAI.12397

41. Toholka R, Wang YS, Tate B, et al. The first Australian Baseline Series: Recommendations for patch testing in suspected contact dermatitis. *Australasian Journal of Dermatology*. 2015;56(2):107-115. doi:10.1111/AJD.12186

42. Fall S, Bruze M, Isaksson M, et al. Contact allergy trends in Sweden – a retrospective comparison of patch test data from 1992, 2000, and 2009. *Contact Dermatitis*. 2015;72(5):297-304. doi:10.1111/COD.12346

43. Wöhrl S, Hemmer W, Focke M, Götz M, Jarisch R. The significance of fragrance mix, balsam of Peru, colophony and propolis as screening tools in the detection of fragrance allergy. *British Journal of Dermatology*. 2001;145(2):268-273. doi:10.1046/J.1365-2133.2001.04345.X

44. Frosch PJ, Duus Johansen J, Schuttelaar MLA, et al. Patch test results with fragrance markers of the baseline series – analysis of the European Surveillance System on Contact Allergies (ESSCA) network 2009–2012. *Contact Dermatitis*. 2015;73(3):163-171. doi:10.1111/COD.12420

45. Simonsen AB, Deleuran M, Mortz CG, Johansen JD, Sommerlund M. Allergic contact dermatitis in Danish children referred for patch testing – a nationwide multicentre study. *Contact Dermatitis*. 2014;70(2):104-111. doi:10.1111/COD.12129

46. L M, M I, M B. Patch Testing with the Swedish Baseline Series in Two Countries. *J Clin Exp Dermatol Res*. 2015;6(5):1-6. doi:10.4172/2155-9554.10000299

47. Mortz CG, Bindslev-Jensen C, Andersen KE. Prevalence, incidence rates and persistence of contact allergy and allergic contact dermatitis in The Odense Adolescence Cohort Study: a 15‐year follow‐up. *British Journal of Dermatology*. 2013;168(2):318-325. doi:10.1111/BJD.12065

48. Duarte IAG, Tanaka GM, Suzuki NM, et al. Patch test standard series recommended by the Brazilian Contact Dermatitis Study Group during the 2006-2011 period. *An Bras Dermatol*. 2013;88(6):1015. doi:10.1590/ABD1806-4841.20132374

49. Uter W, Aberer W, Armario-Hita JC, et al. Current patch test results with the European baseline series and extensions to it from the “European Surveillance System on Contact Allergy” network, 2007-2008. *Contact Dermatitis*. 2012;67(1):9-19. doi:10.1111/J.1600-0536.2012.02070.X

50. Almogren A, Shakoor Z, El Rab MOG, Adam MH. Pattern of patch test reactivity among patients with clinical diagnosis of contact dermatitis: A hospital-based study. *Ann Saudi Med*. 2012;32(4):404-407. doi:10.5144/0256-4947.2012.404

51. Rodrigues DF, Neves DR, Pinto JM, Alves MFF, Fulgêncio ACF. Results of patch-tests from Santa Casa de Belo Horizonte Dermatology Clinic, Belo Horizonte, Brazil, from 2003 to 2010. *An Bras Dermatol*. 2012;87(5):800-803. doi:10.1590/S0365-05962012000500028

52. Thyssen JP, Johansen JD, Linneberg A, Menné T, Engkilde K. The association between contact sensitization and atopic disease by linkage of a clinical database and a nationwide patient registry. *Allergy*. 2012;67(9):1157-1164. doi:10.1111/J.1398-9995.2012.02863.X

53. Yin R, Huang XY, Zhou XF, Hao F. A retrospective study of patch tests in Chongqing, China from 2004 to 2009. *Contact Dermatitis*. 2011;65(1):28-33. doi:10.1111/J.1600-0536.2010.01854.X

54. Landeck L, Schalock P, Baden L, González E. Contact sensitization pattern in 172 atopic subjects. *Int J Dermatol*. 2011;50(7):806-810. doi:10.1111/J.1365-4632.2010.04754.X

55. Cheng S, Cao M, Zhang Y, et al. Time trends of contact allergy to a modified European baseline series in Beijing between 2001 and 2006. *Contact Dermatitis*. 2011;65(1):22-27. doi:10.1111/J.1600-0536.2011.01897.X

56. Isaksson M, Hansson C, Inerot A, et al. Multicentre Patch Testing with Compositae Mix by the Swedish Contact Dermatitis Research Group. *Acta Derm Venereol*. 2011;91(3):295-298. doi:10.2340/00015555-1061

57. Dou X, Zhao Y, Ni C, Zhu X, Liu L. Prevalence of contact allergy at a dermatology clinic in China from 1990-2009. *Dermatitis*. 2011;22(6):324-331. doi:10.2310/6620.2011.11051

58. Beliauskiene A, Valiukeviciene S, Uter W, Schnuch A. The European baseline series in Lithuania: results of patch testing in consecutive adult patients. *Journal of the European Academy of Dermatology and Venereology*. 2011;25(1):59-63. doi:10.1111/J.1468-3083.2010.03688.X

59. Bilcha KD, Ayele A, Shibeshi D, Lovell C. Patch testing and contact allergens in Ethiopia – results of 514 contact dermatitis patients using the European baseline series. *Contact Dermatitis*. 2010;63(3):140-145. doi:10.1111/J.1600-0536.2010.01740.X

60. Garg S, McDonagh AJG, Gawkrodger DJ. Age- and sex-related variations in allergic contact dermatitis to common allergens. *Contact Dermatitis*. 2009;61(1):46-47. doi:10.1111/J.1600-0536.2009.01563.X

61. Wetter DA, Yiannias JA, Prakash A V., Davis MDP, Farmer SA, El-Azhary RA. Results of patch testing to personal care product allergens in a standard series and a supplemental cosmetic series: An analysis of 945 patients from the Mayo Clinic Contact Dermatitis Group, 2000-2007. *J Am Acad Dermatol*. 2010;63(5):789-798. doi:10.1016/j.jaad.2009.11.033

62. Czarnobilska E, Obtulowicz K, Dyga W, Wsolek-Wnek K, Spiewak R. Contact hypersensitivity and allergic contact dermatitis among school children and teenagers with eczema. *Contact Dermatitis*. 2009;60(5):264-269. doi:10.1111/J.1600-0536.2009.01537.X

63. Lam WS, Chan LY, Ho SCK, Chong LY, So WH, Wong TW. A retrospective study of 2585 patients patch tested with the European standard series in Hong Kong (1995–99). *Int J Dermatol*. 2008;47(2):128-133. doi:10.1111/J.1365-4632.2008.03437.X

64. Tudela E, MacPherson C, Maibach HI. Long-term trend in patch test reactions: a 32-year statistical overview (1970–2002), part II. *Cutan Ocul Toxicol*. 2008;27(3):187-202. doi:10.1080/15569520802143436

65. Nardelli A, Carbonez A, Ottoy W, Drieghe J, Goossens A. Frequency of and trends in fragrance allergy over a 15-year period. *Contact Dermatitis*. 2008;58(3):134-141. doi:10.1111/J.1600-0536.2007.01287.X

66. Lindberg M, Edman B, Fischer T, Stenberg B. Time trends in Swedish patch test data from 1992 to 2000. A multi-centre study based on age- and sex-adjusted results of the Swedish standard series. *Contact Dermatitis*. 2007;56(4):205-210. doi:10.1111/J.1600-0536.2006.01063.X

67. Bajaj A, Saraswat A, Mukhija G, Rastogi S, Yadav S. Patch testing experience with 1000 patients. *Indian J Dermatol Venereol Leprol*. 2007;73(5):313-318. doi:10.4103/0378-6323.34008

68. Goon A, Jin T, Teik A, Goon J, Goh CL. Patch Testing of Singapore Children and Adolescents: Our Experience over 18 Years. *Pediatr Dermatol*. 2006;23(2):117-120. doi:10.1111/J.1525-1470.2006.00193.X

69. Heine G, Schnuch A, Uter W, Worm M. Type-IV sensitization profile of individuals with atopic eczema: results from the Information Network of Departments of Dermatology (IVDK) and the German Contact Dermatitis Research Group (DKG). *Allergy*. 2006;61(5):611-616. doi:10.1111/J.1398-9995.2006.01029.X

70. Disphanurat W. Contact allergy in eczema patients in Thammasat University Hospital. *Journal of the Medical Association of Thailand*. 2010;93(SUPPL 7).

71. Bordel-Gómez MaT, Miranda-Romero A, Castrodeza-Sanz J. Epidemiology of Contact Dermatitis: Prevalence of Sensitization to Different Allergens and Associated Factors. *Actas Dermo-Sifiliográficas (English Edition)*. 2010;101(1):59-75. doi:10.1016/S1578-2190(10)70581-3

72. Ertam I, Turkmen M, Alper S. Patch-test results of an academic department in Izmir, Turkey. *Dermatitis*. 2008;19(4). doi:10.2310/6620.2008.08004

73. Zug KA, McGinley-Smith D, Warshaw EM, et al. Contact Allergy in Children Referred for Patch Testing: North American Contact Dermatitis Group Data, 2001-2004. *Arch Dermatol*. 2008;144(10):1329-1336. doi:10.1001/ARCHDERM.144.10.1329

74. Devos SA, Constandt L, Tupker RA, et al. Relevance of Positive Patch-Test Reactions to Fragrance Mix. *Dermatitis*. 2008;19(1):43-47. doi:10.2310/6620.2008.07100

75. Oppel T, Schnuch A. Häufigste Auslöser allergischer Kontaktekzeme. *DMW - Deutsche Medizinische Wochenschrift*. 2006;131(28/29):1584-1589. doi:10.1055/S-2006-947800

76. Magen E, Mishal J, Schlesinger M. Sensitizations to allergens of TRUE test® in 864 consecutive eczema patients in Israel. *Contact Dermatitis*. 2006;55(6):370-371. doi:10.1111/J.1600-0536.2006.00878.X

77. Kashani MN, Gorouhi F, Behnia F, Nazemi MJ, Dowlati Y, Firooz A. Allergic contact dermatitis in Iran. *Contact Dermatitis*. 2005;52(3):154-158. doi:10.1111/J.0105-1873.2005.00545.X

78. Machovcova A, Dastychova E, Kostalova D, et al. Common contact sensitizers in the Czech Republic. Patch test results in 12,058 patients with suspected contact dermatitis*. *Contact Dermatitis*. 2005;53(3):162-166. doi:10.1111/J.0105-1873.2005.00676.X

79. Bruynzeel DP, Diepgen TL, Andersen KE, et al. Monitoring the European standard series in 10 centres 1996–2000. *Contact Dermatitis*. 2005;53(3):146-149. doi:10.1111/J.0105-1873.2005.00541.X

80. Uter W, Hegewald J, Aberer W, et al. The European standard series in 9 European countries, 2002/2003 – First results of the European Surveillance System on Contact Allergies. *Contact Dermatitis*. 2005;53(3):136-145. doi:10.1111/J.0105-1873.2005.00673.X

81. Marinovic-Kulisic S, Lipozenčić J, Ljubojević S, Milavec-Puretić V. Retrospective survey of patch testing at department of dermatology and venerology, zagreb university hospital center in zagreb, croatia. *Acta Dermatovenerol Croat*. 2004;12(4):261-267. Accessed September 16, 2025. https://pubmed.ncbi.nlm.nih.gov/15588559/

82. Piaserico S, Larese F, Recchia GP, et al. Allergic contact sensitivity in elderly patients. *Aging Clin Exp Res*. 2004;16(3):221-225. doi:10.1007/BF03327387/METRICS

83. Goon ATJ, Goh CL. Relevance of positive patch test reactions in patients attending a dermatology tertiary referral centre. *Contact Dermatitis*. 2003;49(5):255-257. doi:10.1111/J.0105-1873.2003.0245.X

84. Boonchai W, Likittanasombat S, Viriyaskultorn N, Kanokrungsee S. Gender differences in allergic contact dermatitis to common allergens. *Contact Dermatitis*. 2024;90(5):458-465. doi:10.1111/COD.14479

85. Pesonen M, Suuronen K, Suomela S, Aalto‐Korte K. Occupational allergic contact dermatitis caused by colophonium. *Contact Dermatitis*. 2019;80(1):9-17. doi:10.1111/cod.13114

86. Färm G. Contact allergy to colophony and hand eczema. *Contact Dermatitis*. 1996;34(2):93-100. doi:10.1111/J.1600-0536.1996.TB02137.X

87. Uter W, Wilkinson SM, Aerts O, et al. Patch test results with the European baseline series, 2019/20—Joint European results of the ESSCA and the EBS working groups of the ESCD, and the GEIDAC. *Contact Dermatitis*. 2022;87(4). doi:10.1111/cod.14170

88. Jensen MB, Isufi D, Larsen CK, Schwensen JFB, Alinaghi F, Johansen JD. Prevalence of Contact Allergy to Neomycin in Dermatitis Patients: A Systematic Review and Meta-Analysis. *Contact Dermatitis*. 2025;93(1):1-15. doi:10.1111/COD.14784

89. Isufi D, Jensen MB, Kursawe Larsen C, Alinaghi F, Schwensen JFB, Johansen JD. Allergens Responsible for Contact Allergy in Children From 2010 to 2024: A Systematic Review and Meta-Analysis. *Contact Dermatitis*. 2025;92(5):327-343. doi:10.1111/COD.14753

90. Owen JL, Vakharia PP, Silverberg JI. The Role and Diagnosis of Allergic Contact Dermatitis in Patients with Atopic Dermatitis. *Am J Clin Dermatol*. 2018;19(3). doi:10.1007/s40257-017-0340-7

91. Hamann CR, Hamann D, Egeberg A, Johansen JD, Silverberg J, Thyssen JP. Association between atopic dermatitis and contact sensitization: A systematic review and meta-analysis. *J Am Acad Dermatol*. 2017;77(1):70-78. doi:10.1016/j.jaad.2017.02.001

92. Isufi D, Jensen MB, Kursawe Larsen C, Alinaghi F, Schwensen JFB, Johansen JD. Allergens Responsible for Contact Allergy in Children From 2010 to 2024: A Systematic Review and Meta-Analysis. *Contact Dermatitis*. 2025;92(5):327-343. doi:10.1111/COD.14753

93. Koh D, Lee BL, Ong HY, Ong CN. Colophony in topical traditional Chinese medicaments. *Contact Dermatitis*. 1997;37(5). doi:10.1111/j.1600-0536.1997.tb02450.x

94. Koh D, Leow YH, Goh CL. Occupational allergic contact dermatitis in Singapore. In: *Science of the Total Environment*. Vol 270. 2001. doi:10.1016/S0048-9697(00)00787-7

95. Chen YX, Gao BA, Cheng HY, Li LF. Survey of Occupational Allergic Contact Dermatitis and Patch Test among Clothing Employees in Beijing. *Biomed Res Int*. 2017;2017. doi:10.1155/2017/3102358

96. Mauro M, Fortina AB, Corradin T, Marino A, Bovenzi M, Filon FL. Sensitization to, and allergic contact dermatitis caused by, colophonium in north-eastern Italy in 1996 to 2016 with a focus on occupational exposures. *Contact Dermatitis*. 2018;79(5). doi:10.1111/cod.13089

97. Isufi D, Christoffer |, Larsen K, et al. Prevalence of Contact Allergy to Rubber Accelerators From the European Baseline Series in Dermatitis Patients: A Systematic Review and Meta-Analysis. *Contact Dermatitis*. 2025;0:1-25. doi:10.1111/COD.70024

98. Uter W, Gefeller O, Mahler V, Geier J. Trends and current spectrum of contact allergy in Central Europe: results of the Information Network of Departments of Dermatology (IVDK) 2007-2018. *Br J Dermatol*. 2020;183(5):857-865. doi:10.1111/BJD.18946

99. Fall S, Bruze M, Isaksson M, et al. Contact allergy trends in Sweden - A retrospective comparison of patch test data from 1992, 2000, and 2009. *Contact Dermatitis*. 2015;72(5). doi:10.1111/cod.12346

100. Widman TJ, Oostman H, Storrs FJ. Allergic contact dermatitis from medical adhesive bandages in patients who report having a reaction to medical bandages. *Dermatitis*. 2008;19(1). doi:10.2310/6620.2008.07053

101. Smith SM, Zirwas MJ. Nonallergic reactions to medical tapes. *Dermatitis*. 2015;26(1):38-43. doi:10.1097/DER.0000000000000098

102. Tam I, Wang JX, Yu J De. Identifying Acrylates in Medical Adhesives. *Dermatitis*. 2020;31(4):E40-E42. doi:10.1097/DER.0000000000000584

103. Hamnerius N, Dahlin J, Bruze M, Nilsson K, Sukakul T, Svedman C. Colophonium-related Allergic Contact Dermatitis Caused by Medical Adhesive Tape Used to Prevent Skin Lesions in Soldiers. *Acta Derm Venereol*. 2023;103. doi:10.2340/actadv.v103.18428

104. Nardelli A, Carbonez A, Drieghe J, Goossens A. Results of patch testing with fragrance mix 1, fragrance mix 2, and their ingredients, and Myroxylon pereirae and colophonium, over a 21-year period. *Contact Dermatitis*. 2013;68(5):307-313. doi:10.1111/COD.12056

105. Lombardo F, Passanisi S, Caminiti L, et al. High Prevalence of Skin Reactions Among Pediatric Patients with Type 1 Diabetes Using New Technologies: The Alarming Role of Colophonium. *Diabetes Technol Ther*. 2020;22(1):53-56. doi:10.1089/DIA.2019.0236

106. Goossens A, Aerts O, Dendooven E. Potential Allergens in Medical Adhesives in Tapes, Wound Dressings, and Ostomy Care: A Scoping Review. *Contact Dermatitis*. Published online 2025. doi:10.1111/COD.70016

107. Dendooven E, Foubert K, Naessens T, et al. Allergic contact dermatitis from (“hypoallergenic”) adhesives containing D-limonene. *Contact Dermatitis*. 2022;86(2):113-119. doi:10.1111/COD.14008

108. de Groot A, Calta E, Ipenburg NA, van Oers EM, Rustemeyer T. Composition of Myroxylon pereirae Resin and Colophonium for Patch Testing. *Contact Dermatitis*. 2025;93(2):108-113. doi:10.1111/COD.14807
