# Supplementary material for: Prevalence of Contact Allergy to Colophonium in Dermatitis Patients: A Systematic Review and Meta‐Analysis
Source: Contact Dermatitis. 2026 Apr 5;95(1):1–16. doi: 10.1111/cod.70153 (PMC13238359; doi:10.1111/cod.70153)
Supplement: Supplementary file 2 — Data S1: PRISMA flow colophonium. [file COD-95-1-s003.docx]

**Identification of studies via databases and registers**

Records removed *before screening*:

- Duplicate removed (n = 977)

Records identified from databases (n = 2300):

- Web of Science (n = 534)
- PubMed (n = 513)
- Embase (n = 1,253)

**Identification**

Records screened (n = 1,323)

Records excluded (n = 1,225)

Reports sought for retrieval (n = 98)

Reports not retrieved

(n = 8)

**Screening**

Reports excluded:

- Conference papers (n = 10)
- Foreign language (n = 3)
- Data already included (n = 2)
- Lack of data (n = 2)

Reports assessed for eligibility

(n = 90)

Studies included in review and meta-analysis (n = 73)

**Included**
